# Interplay of Breast Cancer Resistance Protein (Bcrp/Abcg2), Sex, and Fed State in Oral Pharmacokinetic Variability of Furosemide in Rats

**DOI:** 10.3390/pharmaceutics15020542

**Published:** 2023-02-06

**Authors:** Sheena Sharma, Vijaya Saradhi Mettu, Bhagwat Prasad

**Affiliations:** Department of Pharmaceutical Sciences, Washington State University, Spokane, WA 99202, USA

**Keywords:** furosemide, oral pharmacokinetics, Bcrp, sex-effect, interindividual variability

## Abstract

Poor and variable oral bioavailability of furosemide (FUR) presents critical challenges in pharmacotherapy. We investigated the interplay of breast cancer resistance protein (Bcrp)-mediated transport, sex, and fed state on FUR pharmacokinetics (PK) in rats. A crossover PK study of FUR (5 mg/kg, oral) was performed in Sprague-Dawley rats (3 males and 3 females), alone or with a Bcrp inhibitor, novobiocin (NOV) (20 mg/kg, oral), in both fed and fasted states. Co-administration of NOV significantly increased FUR extent (AUC) and rate (C_max_) of exposure by more than two-fold, which indicates efficient Bcrp inhibition in the intestine. The female rats showed two-fold higher AUC and C_max_, and two-fold lower renal clearance of FUR compared to the male rats. The latter was correlated with higher renal abundance of Bcrp and organic anion transporters (Oats) in the male rats compared to age-matched female rats. These findings suggest that the PK of Bcrp and/or Oat substrates could be sex-dependent in rats. Moreover, allometric scaling of rat PK and toxicological data of Bcrp substrates should consider species and sex differences in Bcrp and Oat abundance in the kidney. Considering that Bcrp is abundant in the intestine of rats and humans, a prospective clinical study is warranted to evaluate the effect of Bcrp inhibition on FUR PK. The potential confounding effect of the Bcrp transporter should be considered when FUR is used as a clinical probe of renal organic anion transporter-mediated drug–drug interactions. Unlike human data, no food-effect was observed on FUR PK in rats.

## 1. Introduction

Furosemide (FUR) is a widely used fast-onset loop diuretic and the first-line treatment option in congestive heart failure, with about 28 million prescriptions in 2019 [1,2]. Like other loop diuretics, FUR increases water excretion by inhibiting the apical sodium-potassium-chloride (Na^+^-K^+^-2Cl^−^) cotransporter in the thick ascending limb of the loop of Henle in kidneys [3]. Although FUR is one of the most successful diuretics, high variability in FUR pharmacokinetics (PK) and bioavailability (10–100%) is a critical challenge in its pharmacotherapy [4].

FUR is a poorly water-soluble (<0.1 mg/mL) and permeable (apparent permeability, ≤2.0 × 10^−6^ cm/s) compound [5,6,7]. FUR is primarily eliminated unchanged in urine through active secretion (fraction excreted unchanged in urine, f_e_ ≈ 65%), with the remaining drug metabolized by UDP-glucuronosyltransferase 1A9 (UGT1A9) [8,9]. FUR is a substrate of organic anion transporters (OAT1 and OAT3) and multidrug resistance-associated protein 4 (MRP4) in the kidneys, which facilitate its basolateral uptake and apical efflux, respectively [10,11,12]. The effect of OATs on the renal elimination of FUR is well studied through clinical drug–drug interactions (DDI) studies. For example, probenecid coadministration substantially decreased OAT-mediated tubular secretion of FUR, leading to its increased systemic exposure [12]. Accordingly, U.S. Food and Drug Administration (FDA) has proposed FUR as a probe substrate of renal OAT1/3 for assessing clinical DDIs during drug development (Shen et al., 2019; U.S. Food and Drug Administration, 2022).

Recently, we and others demonstrated that FUR is also a substrate of breast cancer resistance protein (Bcrp), a transporter expressed on the apical membrane of the epithelium in the small intestine and kidney and on the canalicular membrane of hepatocytes [7,13,14]. Because FUR is commonly co-administered with other drugs to treat cardiovascular and metabolic disorders, the inhibition of Bcrp transport in the intestine can significantly influence its absorption and PK [15]. Moreover, FUR is often used in combination with other loop diuretics (bumetanide), thiazides (trichlormethiazide), or carbonic anhydrase inhibitors (acetazolamide) to overcome the loop diuretic resistance or improve efficacy, which can result a DDI, as these drugs are known Bcrp inhibitors [16,17,18]. Since Bcrp is expressed in other tissues such as kidneys, the brain, and placenta [19,20,21], transport inhibition can affect systemic or local tissue concentrations of FUR. Although we have demonstrated that the substrates of efflux transporters are influenced by food [22], such mechanisms are not explored for FUR. The primary aim of this study was to quantify the effect of Bcrp inhibition by novobiocin (NOV) on oral absorption of FUR in a rat model in fed and fasting states. Because renal Bcrp and Oat abundance is higher in male compared to female rats [23], we also characterized the confounding effect of sex on FUR PK. The in vivo examination of the role of the intestinal Bcrp efflux transporter and sex on FUR PK in rats provides directions for prospective evaluations of FUR PK and DDI in humans.

## 2. Materials and Methods

### 2.1. Chemicals, Reagents, and Software

FUR and NOV sodium were purchased from Sigma-Aldrich (St. Louis, MO, USA). FUR-d5 was purchased from Toronto Research Chemicals (Toronto, ON, Canada). LC-MS grade acetonitrile, methanol, ethanol, chloroform, and formic acid were purchased from Fisher Scientific (Fair Lawn, NJ, USA). IV catheters (22-gauge, diameter 0.9 × 25 mm, metal needle) and 1 mL disposal syringes were purchased from Becton, Dickinson and Co (Franklin Lakes, NJ, USA). Surgical tape, chlorhexidine, betadine, and heparin were purchased from a local pharmacy (Spokane, WA, USA). Isoflurane, heating lamp, and 70% ethanol were procured from the Program of Laboratory Animal Resources Vivarium of the Washington State University Health Sciences (Spokane, WA, USA). Ammonium bicarbonate (98% pure), bovine serum albumin, dithiothreitol, iodoacetamide, trypsin, and stable isotope-labeled peptide were purchased from Thermo Fisher Scientific (Rockford, IL, USA). MATLAB (Natick, MA, USA), BioRender (Toronto, ON, Canada), GraphPad (San Diego, CA, USA), and Microsoft Office Excel and PowerPoint (Redmond, WA, USA) were used to create the figures and analyze data.

### 2.2. Rat Pharmacokinetic Study

The animal PK study was performed in accordance with the recommendations in the Guide for the Care and Use of Laboratory Animals of National Institutes of Health. The experimental protocols were approved by the Washington State University Institutional Animal Care and Use Committee (IACUC; approval number, 6750). Blood samples were collected by tail vein cannulation, which was performed under anesthesia, and all efforts were made to minimize pain and discomfort.

Three male and three female Sprague-Dawley rats (age, 9–12 weeks; body weight, 225–350 g) were procured from Charles River Laboratories (Hollister, CA, USA). The rats were housed 2–3 per cage in a specific-pathogen-free facility with controlled light (14/10 h, light/dark cycle), temperature (20–26 °C), and humidity (50 ± 20%). Food and water were provided ad libitum prior to the study.

FUR and NOV were dissolved in 100 mM phosphate-buffered saline (PBS) (pH 7.0, adjusted with 0.1 N sodium hydroxide) to achieve 1 mg/mL and 8 mg/mL free base concentrations, respectively. NOV (20 mg/kg) was administered about 2–5 min prior to FUR (5 mg/kg) through oral gavage to the rats. 

**Figure 1:**
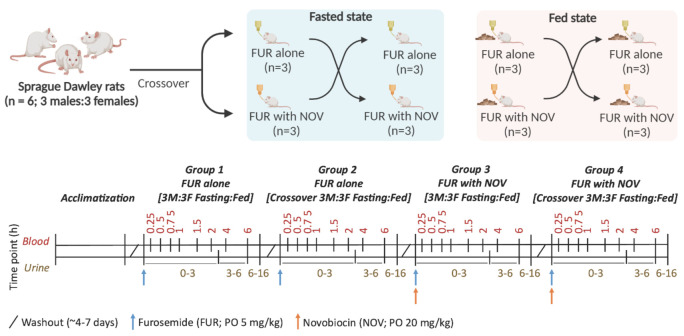
Schematic of rat pharmacokinetic study design to investigate the effect of Bcrp inhibition, sex, and fed state in oral absorption and disposition of furosemide (FUR). Six rats (3 males and 3 females) were used in a cross-over fashion, and the study was conducted in the following four groups: (1) FUR alone in fed state, (2) FUR alone in fasting state, (3) FUR with novobiocin (NOV) in fed state, and (4) FUR with NOV in fasting state. Blood samples were collected from 0 to 6 h and urine was collected at 0–3 h, 3–6 h, and 6–16 h time intervals. Because blood samples could not be collected at certain timepoints from one female rat due to a technical difficulty in the tail vein catheterization, only five rats were included in the data analysis.

A crossover PK study of FUR was performed in the following four groups: (1) FUR alone in fed, (2) FUR alone in fasting, (3) FUR with NOV in fed, and (4) FUR with NOV in fasting conditions (Figure 1). For groups 2 and 4, the rats were fasted between 4 h prior to dosing and 2 h post-dose with free access to water. Briefly, the rats were anesthetized using isoflurane and the tail vein was dilated through brief heating and sterilization with 70% ethanol. The rats were then placed in a restrainer and the lateral tail vein was located to insert a catheter, and heparinized saline was used to maintain patency of the catheter following each sampling. The blood samples (25 µL) were collected in duplicate in 1 mL tubes and mixed with an equal volume of water containing heparin (30 IU/mL containing 0.05% formic acid) at pre-dose, 0.25, 0.5, 0.75, 1, 1.5, 2, 4, and 6 h. Urine samples were collected between 0–3, 3–6, and 6–16 h and mixed with 5% deionized water containing 0.05% formic acid. The collected blood and urine samples were stored in a −80 °C freezer until liquid chromatography–tandem mass spectrometry (LC-MS/MS) analysis.

### 2.3. Analysis of Blood and Urine Samples

A mix of stock solutions of FUR and NOV was serially diluted into twelve working standards in methanol (0.012–25 µg/mL and 0.0978–100 µg/mL, respectively). Similarly, three quality control (QC) working standards were prepared at low (0.048 and 0.39 µg/mL), medium (1.56 and 3.12 µg/mL), and high (12.5 and 50 µg/mL) FUR and NOV concentrations, respectively. The working standard solution (5 µL) was spiked into 45 µL of a blank biological matrix (blood: water, 50:50 *v*/*v*) to prepare matrix-matched calibration curve standards and QC samples. Next, 300 µL of acetonitrile containing FUR-d5 (as internal standard; 300 ng/mL) was added to 50 µL of the calibration curve standards, QC samples, and rat blood samples to precipitate blood proteins. The samples were vortex mixed followed by centrifugation at 16,000× *g* (4 °C) for 10 min. The supernatant (50 µL) was collected and transferred to an LC vial for analysis using an M-class Waters UPLC system coupled with a Waters Xevo^®^ TQ-XS MS instrument connected by a standard electrospray ionization source. One μL of the sample was injected and LC separation was achieved using an Acquity UPLC HSS T3 column (100Å, 1.8 µm, 1 mm × 100 mm) and mobile phase (A: water with 0.1% formic acid and B: acetonitrile with 0.1% formic acid; 50 μL/min flow) with a gradient program: 0.0–1.5 min (5% B), 1.5–3.5 min (5–70% B), 3.5–5 min (70–90% B), 5–7.5 min (90% B), and 7.5–7.6 min (90–5% B), followed by 5% B until 8.5 min. The data acquisition was performed in the multiple reaction monitoring (MRM) mode using the following parameters: FUR (*m*/*z* 329.0 → 285.0 and 205.0; collision energy (CE) 15 and 20 eV, respectively), FUR-d5 (*m*/*z* 334.1 → 290.0; CE 10 eV), and NOV (*m*/*z* 613.39 → 189.1 and 369.1; CE 15 and 5 eV, respectively). The cone voltage (CV) was 25 V.

Similarly, 25 µL of urine sample (diluted 10-fold with water containing 0.1% formic acid) was mixed with 75 µL of acetonitrile containing 100 ng/mL of FUR-d5. The sample was vortex mixed followed by centrifugation at 16,000× *g* for 10 min (4 °C). The supernatant (50 µL) was collected and transferred to an LC vial for analysis by LC-MS/MS. The LC conditions and MRM transitions for FUR and NOV were similar to that described for the blood samples except that an injection volume of 2 µL was used and a longer gradient was employed to avoid a matrix effect, i.e., 0.0–1 min (5% B), 1–5 min (5–55% B), 5–8.5 min (55–70% B), 8.5–10 min (70–90% B), 10–12.5 min (90–95% B), and 12.5–12.6 min (95–5% B), followed by 5% B until 13.5 min. 

The calculation of PK endpoints (area under the blood concentration–time profile curve, AUC, and peak blood concentration, C_max_) was performed using noncompartmental PK analysis by MATLAB R2021b software (Natick, MA). The data were expressed as mean ± standard deviation (SD). The renal clearance (CL_R_) was calculated using the following equation [24].

(1) $${\mathrm{CL}}_{R}=\frac{A_{e\;0-6\;h}}{{\mathrm{AUC}}_{0-6\;h}}\;$$

where, A_e_ is the cumulative amount of drug excreted unchanged in the urine between 0 to 6 h.

AUC and C_max_ were compared between different groups using (i) Student’s *t*-test for FUR alone versus FUR with NOV, male versus female rats, and fed versus fasted states, or (ii) analysis of variance (ANOVA) followed by Tukey’s multiple comparisons test to compare the effect of Bcrp inhibition, sex, and fed state. Bcrp inhibition and food-effect were analyzed using paired analysis, whereas the sex-effect was evaluated by an unpaired analysis.

### 2.4. Quantification of Bcrp Protein Abundance in Rat Tissues 

Total Membrane Protein Isolation: The total membrane protein was extracted from rat kidney, jejunum, and ileum tissue homogenates using the Mem-PER^TM^ Plus kit (Thermo Fisher Scientific; Rockford, IL, USA), as previously described [25]. Briefly, tissue (~100 mg) was homogenized in the ice-cold homogenization buffer containing 0.5% protease inhibitor using a bead homogenizer (VWR; Radnor, PA, USA). The tissue homogenate (950 µL) was centrifuged at 1000× *g* for 10 min (4 °C) to remove the nuclear fraction. The supernatant was transferred to a 1.5 mL tube and centrifuged at 16,000× *g* for 15 min. The supernatant (i.e., cytosolic fraction) was discarded and the pellet was resuspended with 500 µL of the homogenization buffer and incubated in a shaking rotor at 300 rpm for 30 min (4 °C). The sample was centrifuged at 16,000× *g* for 15 min, and the supernatant (i.e., cytosolic wash fraction) was removed. The pellet was resuspended in 500 µL of the membrane solubilization buffer (1 mL of 4% sodium dodecyl sulfate and 5 µL of protease inhibitor cocktail per ml of the solubilization buffer), followed by incubation for 60 min at 300 rpm (15 °C). The solubilized total membrane protein fraction was stored at −80 °C before digestion.

Bcrp Quantification by LC-MS/MS: An optimized targeted LC-MS/MS methodology was used to selectively quantify Bcrp protein abundance in rat tissues [26]. Briefly, 80 μL of the tissue sample (1 mg/mL total protein) was mixed with 30 μL ammonium bicarbonate (100 mM) and 20 μL of bovine serum albumin (0.02 mg/mL). Proteins were denatured and reduced with 10 μL of dithiothreitol (250 mM) through gentle shaking at 300 rpm for 10 min (95 °C). The sample was cooled at room temperature for 10 min and the denatured protein was alkylated with 10 μL iodoacetamide (100 mM) in the dark for 30 min. One milliliter of ice-cold acetone was added to the sample, followed by vortex-mixing and incubation at −80 °C for 30 min. The sample was then centrifuged at 16,000× *g* (4 °C) for 10 min and the protein pellet was collected and dried at the room temperature for 30 min and washed with 500 μL ice-cold methanol, followed by centrifugation at 16,000× *g* (4 °C) for 10 min. The pellet was collected and dried at room temperature for 30 min and resuspended in 60 μL ammonium bicarbonate buffer (50 mM, pH 7.8). Finally, the reconstituted protein sample was digested by adding 20 μL of trypsin (50 protein:1 trypsin) and incubated for 16 h (37 °C). The sample was centrifuged at 1000× *g* (4 °C) for 1 min and kept in a −20 °C freezer for 5 min. The reaction was quenched by adding 10 μL of the peptide internal standard cocktail (prepared in a solution of 80% acetonitrile and water containing 0.5% formic acid) and 5 μL of 5% formic acid in water. The sample was vortex mixed and centrifuged at 16,000× *g* for 10 min. The supernatant was collected in an LC-MS vial for the analysis, which was achieved using the parameters provided in Tables S1 and S2. The samples were digested and analyzed in triplicate. The peptides were separated using an HSS T3 C18 column (1.8 μm, 1.0 × 100 mm). The proteotypic peptide of Bcrp (SSLLDVLAAR) was quantified in the digested samples using a previously reported protocol [26]. The relative protein abundance of Bcrp was quantified and a stable-labeled peptide containing [^13^C_6_ ^15^N_4_]-arginine served as the internal standard. The proteomics data were analyzed using Skyline 19.1 (University of Washington, Seattle, WA, USA).

## 3. Results

### 3.1. Effect of Bcrp Inhibition on Pharmacokinetics of Furosemide

**Figure 2:**
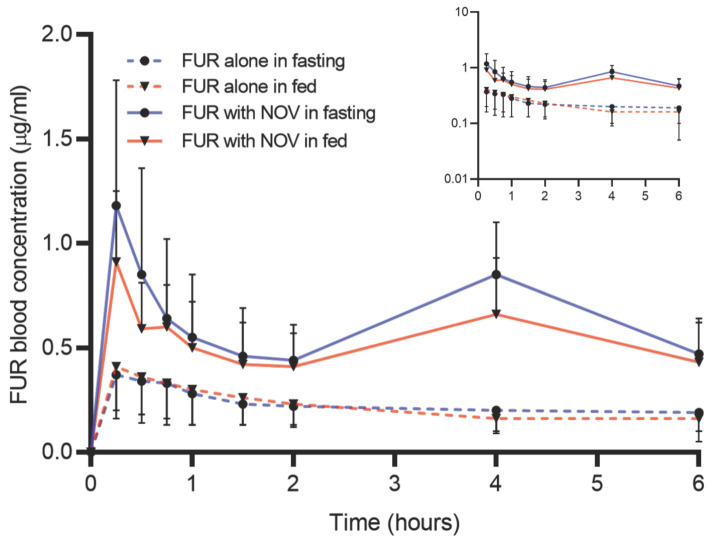
Furosemide (FUR) blood concentrations in fed and fasted states after 5 mg/kg PO dose, alone or with 20 mg/kg PO novobiocin (NOV) in the rats (n = 6; 3 males and 3 females). The double-peak in FUR PK when co-administered with NOV correlated with the higher NOV concentration at the 4-h time-point. The inset shows the semi-log curve.

**Figure 3:**
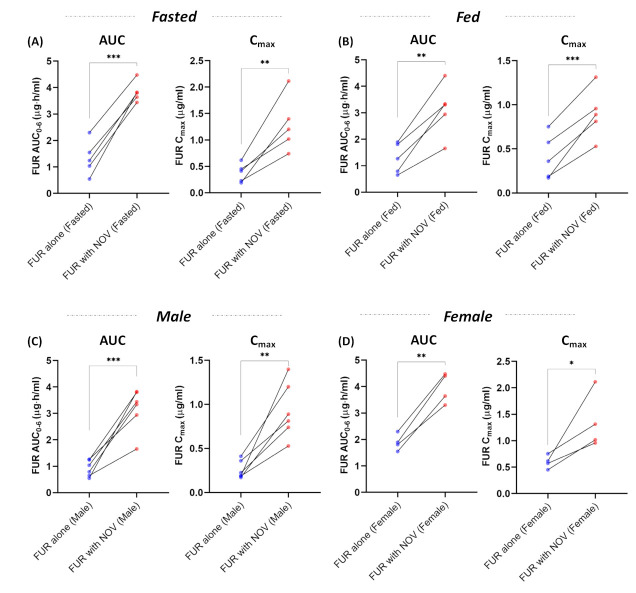
Effect of Bcrp inhibition on the AUC and C_max_ of furosemide (FUR) in the fasted and fed states (**A**,**B**) and in male and female rats (**C**,**D**) (n = 5; 3 males and 2 females). Coadministration of novobiocin (NOV) increased the AUC and C_max_ by >2-fold (*p*-value < 0.05) of FUR, irrespective of food and sex. The symbols represent individual data points and the lines connect the paired samples. Data were compared by paired Student’s *t*-test (* *p*-value ≤ 0.05, ** *p*-value ≤ 0.01, and *** *p*-value ≤ 0.001).

The PK profiles of FUR were analyzed with and without NOV in both fed and fasted states (Figure 2). In the fasted state, coadministration of NOV significantly increased FUR AUC and C_max_ by 3-fold (*p*-value < 0.001) (Figure 3A). Similarly, in the fed state, FUR AUC and C_max_ were 2-fold (*p*-value < 0.05) higher following NOV coadministration compared to the FUR alone condition (Figure 3B). Irrespective of sex, FUR AUC and C_max_ were higher following the coadministration of NOV. In the male rats, FUR AUC and C_max_ were increased by 3- to 4-fold (*p*-value < 0.001) when co-administered with NOV (Figure 3C). The female rats showed a 2-fold (*p*-value < 0.05) increase in FUR AUC and C_max_ with NOV (Figure 3D). Co-administration of NOV showed no effect on CL_R_ of FUR (Figure S1).

### 3.2. Effect of Sex on Pharmacokinetics of Furosemide and Novobiocin

**Figure 4:**
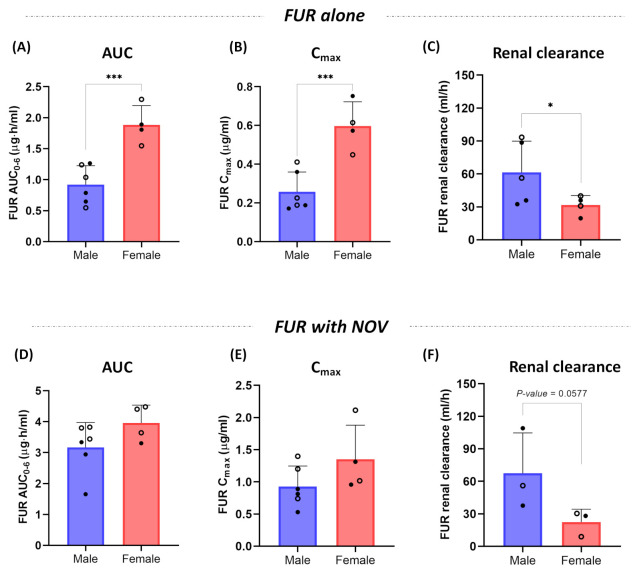
Effect of sex on the AUC, C_max_, and renal clearance of furosemide (FUR) alone (**A**–**C**) and with novobiocin (NOV) (**D**–**F**) in the rats. The female rats showed ~2-fold higher AUC and C_max_, and 2-fold lower renal clearance compared to the male rats. However, no sex-effect was observed in FUR pharmacokinetics when FUR was co-administered with NOV. Because no food-effect was observed, fed (closed circle) and fasted (open circle) data for the male and female rats were included as separate data points (n = 5; 3 males and 2 females in fed and fasting states, respectively). The symbols represent individual data points. Data were compared by unpaired Student’s *t*-test (* *p*-value ≤ 0.05, *** *p*-value ≤ 0.001; *p*-value > 0.05 was considered not significant).

The female rats showed ~2-fold higher (*p*-value < 0.001) FUR AUC and C_max_ compared to the male rats (Figure 4A,B). Consistent with FUR blood levels, the male rats showed 2-fold higher FUR CL_R_ compared to the female rats, i.e., 61.3 and 31.6 mL/h, respectively, (Figure 4C). No sex-effect was observed on the AUC, C_max_, or CL_R_ of FUR when co-administered with NOV (Figure 4D–F).

### 3.3. Cumulative Effect of Bcrp Inhibition, Sex, and Fed State on Pharmacokinetics of Furosemide

The AUC of FUR was higher with NOV in both the fasted and fed male rats when compared to FUR alone in the fasted and fed male rats (Figure S2A). Similarly, FUR C_max_ was higher in fasted male rats when co-administered with NOV compared to the fed male rats given FUR alone (Figure S2B). The PK of FUR in the fed state was not different compared to the fasted state when dosed alone or with NOV (Figure S3). The male and female rats showed comparable AUC and C_max_ of NOV (Figure S4A,B). Although the CL_R_ of NOV in the male rats was higher compared to the female rats (2.3 and 0.5 mL/h, respectively), this difference was not statistically significant. Similarly, no food-effect was observed on the PK of NOV in the fed state (Figure S4D,E).

**Figure 5:**
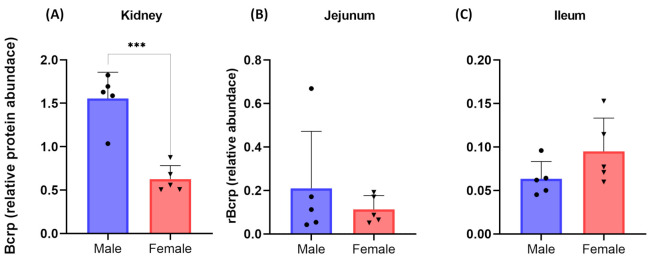
Tissue-specific effect of sex on Bcrp protein abundance in the rats (n = 10; 5 males and 5 females). Bcrp abundance (normalized by the total membrane protein) was 2-fold higher in the male rat kidney (**A**). Bcrp abundance was comparable in the intestinal segments of male versus female rats (**B**,**C**). The symbols represent individual data points. Data were compared using unpaired Student’s *t*-test (*** *p*-value ≤ 0.001).

To further explain sex-dependent PK of FUR, we quantified Bcrp abundance in rat kidney and intestinal segments. The male rats showed 2-fold higher (*p*-value < 0.05) abundance of Bcrp in the kidney (Figure 5A). However, Bcrp abundance was not statistically different in the intestinal segments (Figure 5B,C).

## 4. Discussion

FUR exhibits significant intra- and inter-individual variability in its PK with up to ~10-fold difference in the bioavailability [4]. The primary objective of the study was to investigate the effect of Bcrp inhibition on the absorption of FUR in the presence of a selective Bcrp inhibitor (NOV), whereas sex and fed state were considered covariates. Similar to humans, Bcrp expression in rats is abundant in the intestinal segments, but only rat kidneys express Bcrp to a detectable level. For example, the levels of Bcrp in rat and human kidney are 4.5 pmol/mg protein and below limit of quantification (0.09 pmol/mg protein), respectively [23,27,28,29]. This suggests that the effect of Bcrp inhibition on the bioavailability of its substrates can be investigated using the rat model; however, rat is not a good species for translating renal elimination of Bcrp substrates. In this study, we observed that Bcrp inhibition by NOV leads to a more than two-fold increase in FUR systemic exposure with no effect on the CL_R_, confirming that Bcrp inhibition is intestine-specific. The CL_R_ estimation based on the AUC_0-6_ data might not represent the full PK profile of furosemide. Nevertheless, the primary goal of this study was to investigate the effect of Bcrp inhibition on the absorption of furosemide, which can be reliably tested using 6-h AUC data. In addition, CL_R_ using the midpoint approach [24,30] (Figure S5) showed a similar trend (male > female rats) on the effect of sex on CL_R_. Further, the effect of Bcrp inhibition on FUR PK was more pronounced in the male rats compared to the female rats. Although the oral dose of NOV was 20 mg/kg, the body weight of the age-matched male and female rats was significantly different (~500 g versus 250 g, respectively), which is a common phenomenon [31]. Therefore, the body weight normalized NOV dose was almost twice as high in the male rats, which could likely lead to greater Bcrp inhibition in the male rats.

Considering the clinical relevance of Bcrp inhibition, the FDA recommends investigating the DDI potential of Bcrp substrates with co-administered drugs [32]. Bcrp inhibition has been shown to increase the AUC and C_max_ of Bcrp substrates such as rosuvastatin and sulfasalazine in the clinic [33,34,35,36]. However, the effect of Bcrp inhibition on FUR is not well-characterized in humans. The present study provides proof-of-concept rat data that support the need for investigating the DDI potential of FUR with Bcrp inhibitors. In particular, polypharmacy increases the incidences of DDI, which is likely in geriatric populations and in patients with chronic ailments [37,38]. FUR is commonly prescribed with calcium channel blockers for hypertension that are known Bcrp inhibitors, such as nicardipine and nitrendipine [39,40,41]. Further, ABCG2 (Bcrp gene) is highly polymorphic with a common single nucleotide polymorphism (SNP; 421C>A), which is associated with decreased Bcrp content and activity, and thus warrants clinical investigation of Bcrp substrates such as FUR [19,42,43]. Further, intestinal Bcrp can be inhibited by dietary components such as quercetin and curcumin [36,44].

In this study, the female rats showed two-fold higher AUC and C_max_ of FUR compared to the male rats. These data corroborate with Bcrp abundance in rat kidneys, as presented in Figure 5 and reported previously [23,45]. Because Bcrp levels in human kidneys are not detectable [46], the species-difference in Bcrp activity and content should be considered when translating preclinical data to humans. The sex-differences in FUR PK observed in this study are corroborated by the higher efficacy data reported in female rats [47]. A clinical study showed higher bioavailability of FUR in females compared to males; however, the sex-effect was diminished in the fed state [48]. Other Bcrp substrate drugs are also shown to have higher systemic clearance in men versus women, e.g., topotecan [49], methotrexate [50,51], doxorubicin [52], and epirubicin [53]. The current understanding is that OATs play a rate-determining role in FUR systemic concentration, and the sex-effect on FUR PK in rats can be partially attributed to 1.3-fold higher OAT levels in male rats [23,54]. In addition, consistent with 2-fold higher renal Bcrp abundance in male versus female rats observed in this study, the published report also suggested higher (1.7-fold) Bcrp in the male rat kidneys [23]. Therefore, the sex-specific renal clearance of FUR could be a cumulative effect of sex-dependent OAT and Bcrp abundances. However, we cannot rule out the primary renal transporter contributing to the sex-specific PK of FUR using the existing tools of renal transport investigation.

Despite conflicting reports of food-effect on FUR PK, majority of the studies reveal a negative food-effect for FUR in humans [55,56,57,58,59,60,61]. For example, a 30% decrease in FUR bioavailability is reported in humans after food [60]. However, we did not find a significant food-effect in rats, which is consistent with the reported study in rats [62]. This discrepancy is perhaps because of the higher theoretical luminal concentration of FUR in the rats, considering the higher dose volume (~1.5–3 mL) of FUR compared to the total luminal volume (~3.4 mL) [63]. The higher theoretical luminal drug concentration might have presented solubility-limited absorption instead of permeability-limited absorption in the rats [22]. We previously explained that the permeability-limited drugs are prone to negative food-effect due to the potential interplay of prolonged gastric emptying time in the fed state, leading to an increased efficiency of efflux transporters [22].

Unlike other Bcrp inhibitors that also interact with P-glycoprotein, the selectivity of NOV towards Bcrp is well-characterized [64,65,66,67]. Suzuki et al. showed that coadministration of NOV significantly increased the AUC and C_max_ of sulfasalazine in rats after oral administration by 3.2- and 5.9-fold, respectively [68]. However, the systemic clearance of sulfasalazine following intravenous dosing was not influenced in the study. These results confirm that NOV selectively inhibits intestinal Bcrp-mediated efflux without affecting Bcrp activity in other organs. However, NOV is also reported as an OAT inhibitor [69]. To address this potential confounding effect, we selected an oral NOV dose of 20 mg/kg to achieve a concentration (<5 µM) that would selectively inhibit Bcrp in the rats without affecting OATs. This assumption was based on the half maximal inhibitory concentration (IC_50_) values of NOV for human Bcrp, OAT1, and OAT3, which are reported as 1.4, 34.8, and 5 µM, respectively [65,67,69]. The inhibition of renal OATs in rats is unlikely by NOV, as its unbound C_max_ is 35- and 244-fold lower than the IC_50_ values for OAT3 and OAT1, respectively.

Although the study was adequately powered (80% power and 0.05 alpha) to estimate the minimum number of rats for achieving significance, future studies with replicates can be performed to support the findings. Nevertheless, the species and sex differences in the abundance of Bcrp in the kidney should be considered when using rat PK and toxicological data to predict the effects of Bcrp substrates in humans using allometric scaling. As Bcrp is abundant in the intestine of both rats and humans, clinical studies are needed to assess the impact of Bcrp inhibition on the PK of FUR in humans.

## 5. Conclusions

This is the first study to demonstrate a significant DDI between FUR and a Bcrp inhibitor, and suggests that rats are a sensitive model for evaluating the effect of intestinal Bcrp inhibition on its substrates. However, rat is not a good species for translating renal elimination of Bcrp substrates because of the large species differences in human versus rat Bcrp abundance. Further, the study indicated the potential effect of sex on FUR PK. Although additional disposition mechanisms may partially explain sex-differences in toxicological studies, sex-dependent Bcrp abundance in rats should be considered in allometric scaling. The current study supports the importance of studying drug PK, efficacy, and toxicity in both sexes. As FUR has been proposed as a probe substrate of renal OAT1/3 for assessing clinical DDIs during drug development [12,70], it is important to consider the potential confounding effect of intestinal Bcrp and sex on FUR PK.

## Data Availability

Data are contained within the article or Supplementary Materials.

## Supplementary Materials

The following supporting information can be downloaded at: https://www.mdpi.com/article/10.3390/pharmaceutics15020542/s1, Figure S1: Effect of novobiocin (NOV) on furosemide (FUR) renal clearance in the rats, Figure S2: Cumulative effect of Bcrp inhibition by NOV, sex and fed state on the AUC (A) and C_max_ (B) of furosemide (FUR) in the rats (n = 5; 3 males and 2 females in fed and fasting states, respectively), Figure S3: Effect of food on the AUC and C_max_ of furosemide (FUR) administered alone (A,B) and with novobiocin (NOV; C,D) in the rats (n = 5; 3 males and 2 females), Figure S4: Effect of sex (A,C) and fed state (D,E) on the AUC, C_max_ and renal clearance of novobiocin (NOV); Figure S5: Renal clearance of furosemide (FUR) alone (A), and with novobiocin (NOV) (B), calculated using mid-point approach (${\mathrm{CL}}_{R}=\frac{{\mathrm{Ae}}_{0-3}/\Delta t}{C_{1.5}};\;$ Table S1: Chromatographic conditions for the separation of proteotypic peptides of rat Bcrp, Table S2: List of proteotypic peptides used for targeted protein quantification of Bcrp and bovine serum albumin (BSA; internal standard), and their multiple reaction monitoring (MRM) parameters for LC-MS/MS analysis.

## Abbreviations

AUC	area under the blood concentration-time profile curve
Bcrp	breast cancer resistance protein
CL_R_	renal clearance
C_max_	peak blood concentration
DDI	drug-drug interactions
FUR	Furosemide
LC-MS/MS	liquid chromatography-tandem mass spectrometry
NOV	Novobiocin
OAT	organic anion transporters
PK	Pharmacokinetics
