# Supplementary material for: Interplay of Breast Cancer Resistance Protein (Bcrp/Abcg2), Sex, and Fed State in Oral Pharmacokinetic Variability of Furosemide in Rats
Source: Pharmaceutics. 2023 Feb 6;15(2):542. doi: 10.3390/pharmaceutics15020542 (PMC9968170; doi:10.3390/pharmaceutics15020542)
Supplement: Supplementary file 1 [file pharmaceutics-15-00542-s001.zip › pharmaceutics-2172804-supplementary.pdf]

Supplementary Materials: Interplay of Breast Cancer Resistance Protein (Bcrp/Abcg2), Sex, and Fed State in Oral Pharmacokinetic Variability of Furosemide in Rats

Sheena Sharma †, Vijaya Saradhi Mettu † and Bhagwat Prasad *

Department of Pharmaceutical Sciences, Washington State University, Spokane, WA 99202, USA

* Correspondence: bhagwat.prasad@wsu.edu; Tel.: +1-(509)-358-7739; Fax: +1-509-368-6561

† These authors contributed equally to this work.

Table S1. Chromatographic conditions for the separation of proteotypic peptides of rat Bcrp

Analytical column: Acquity UPLC HSS T3 column (100 Å, 1.8 µm, 1 x 100 mm)<br>Column Temperature: 40 °C			
Injection volume: 1 µL			
**LC gradient program**			
Time (min)	Flow Rate (µL)	A (Water with 0.1% formic acid, %)	B (Acetonitrile with 0.1% formic acid, %)
0	50	95	5
3	50	95	5
8	50	87	13
20	50	55	45
21.5	50	20	80
22.5	50	20	80
23	50	95	5
28	50	95	5

Table S2. List of proteotypic peptides used for targeted protein quantification of Bcrp and bovine serum albumin (BSA; internal standard), and their multiple reaction monitoring (MRM) parameters for LC-MS/MS analysis. R and K (shown in bold) was labeled by ^{13}C and ^{15}N in the stable-labeled peptide.

Protein	Peptide sequence	Peptide label	Parent ion (m/z)	Product ion (m/z)	Collision energy (eV)	Cone voltage (V)
Bcrp	SSLLDVLAA**R**	Light	522.81	317.19	18	35
			522.81	430.28	18	35
			522.81	529.35	18	35
			522.81	644.37	18	35
			522.81	757.46	18	35
		Heavy	527.81	327.20	18	35
			527.81	440.29	18	35
			527.81	539.35	18	35
			527.81	654.38	18	35
			527.81	767.46	18	35
BSA	AEFVEVT**K**	Light	461.75	476.272	16	35
			461.75	575.34	16	35
			461.75	722.41	16	35
		Heavy	465.76	484.29	16	35
			465.76	583.35	16	35
			465.76	730.42	16	35

Figures

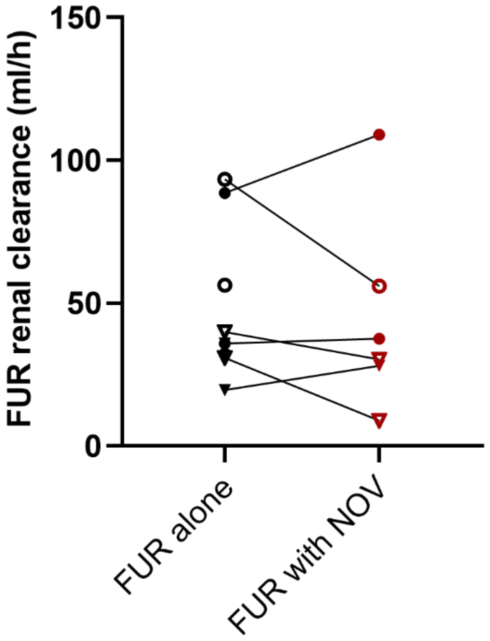

Figure S1. Effect of novobiocin (NOV) on furosemide (FUR) renal clearance in the rats. NOV co-administration showed no effect on the renal clearance of FUR. The symbols represent individual data points (circle, male rats; triangle, female rats) and the lines connect the paired samples. Because no food-effect was observed, fed (closed symbol) and fasted (open symbol) data for the male and female rats were included as separate data points (n = 5; 3 males and 2 females in fed and fasting states, respectively). Data were compared by paired Student's t-test (*P-value* > 0.05 was considered not significant).

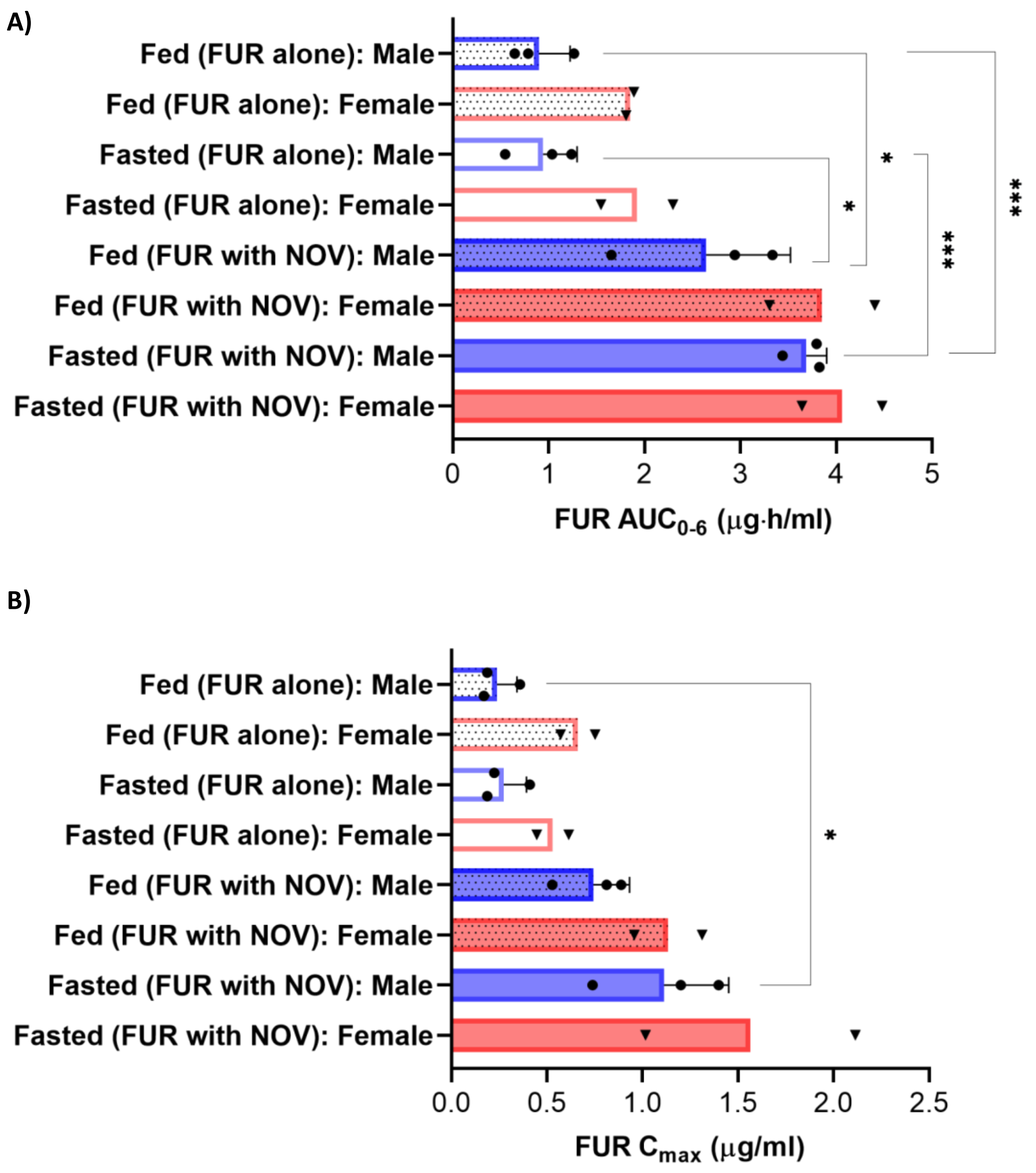

Figure S2. Cumulative effect of Bcrp inhibition by novobiocin (NOV), sex and fed state on the AUC (A) and C_{max} (B) of furosemide (FUR) in the rats (n = 5; 3 males and 2 females in fed and fasting states, respectively). Data were compared by one-way ANOVA followed by Tukey's multiple comparison test (**P-value* ≤ 0.05, ****P-value* ≤ 0.001), except for the female rats (n=2).

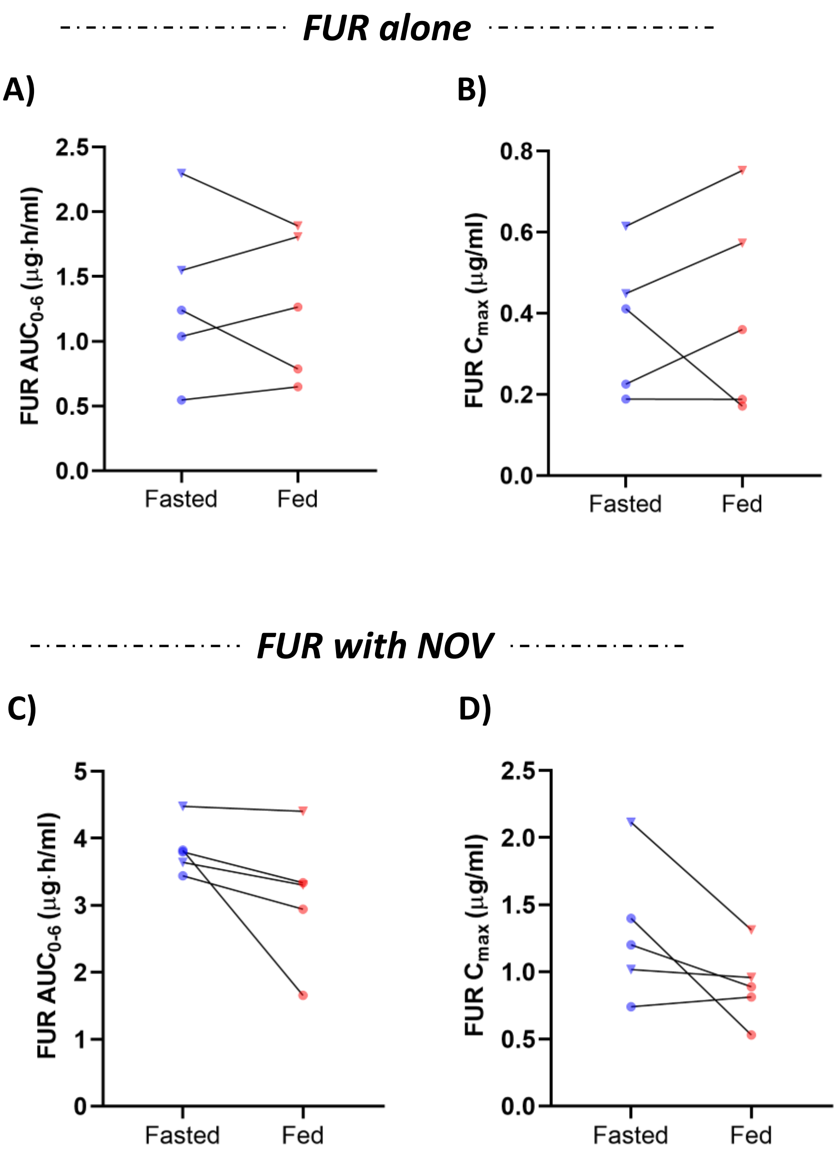

Figure S3. Effect of food on the AUC and C_{max} of furosemide (FUR) administered alone (A-B) and with novobiocin (NOV; C-D) in the rats (n = 5; 3 males and 2 females). No food-effect was observed on the AUC and C_{max} of FUR in the rats. The symbols represent individual data points (circle, male rats; triangle, female rats) and the lines connect the paired samples. Data were compared by paired Student's t-test (*P-value* > 0.05 was considered statistically not significant).

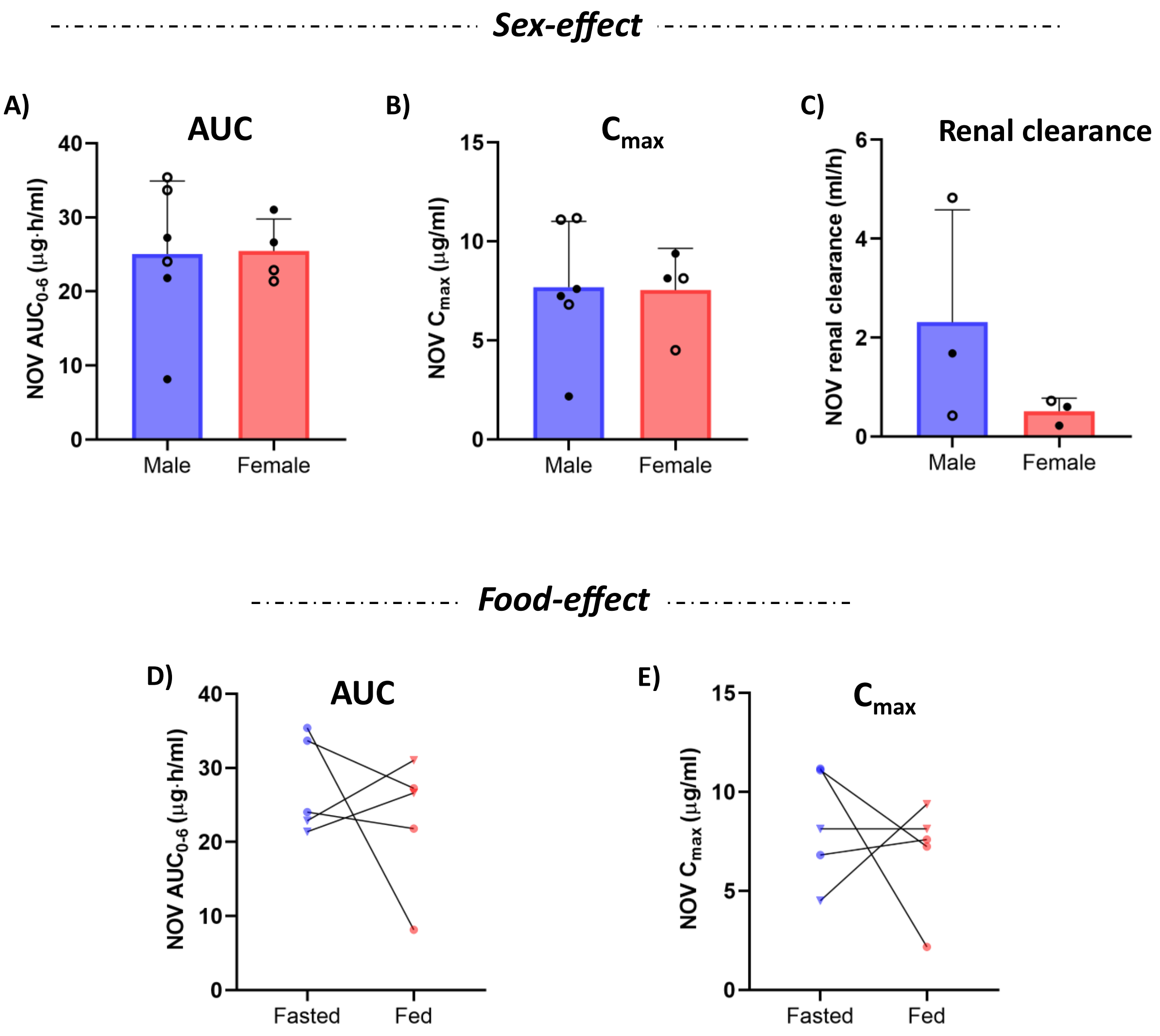

Figure S4. Effect of sex (A-C) and fed state (D- E) on the AUC, C_{max} and renal clearance of novobiocin (NOV). Because no food-effect was observed, fed (closed circle) and fasted (open circle) data for the male and female rats were included as separate data points (A-C). The symbols represent individual data points (n = 5; 3 males and 2 females, represented by circle and triangle symbols, respectively) and the lines connect the paired samples (D-E). Sex-and food-effect data were compared using unpaired and paired Student's t-tests, respectively (*P-value* > 0.05 was considered not significant).

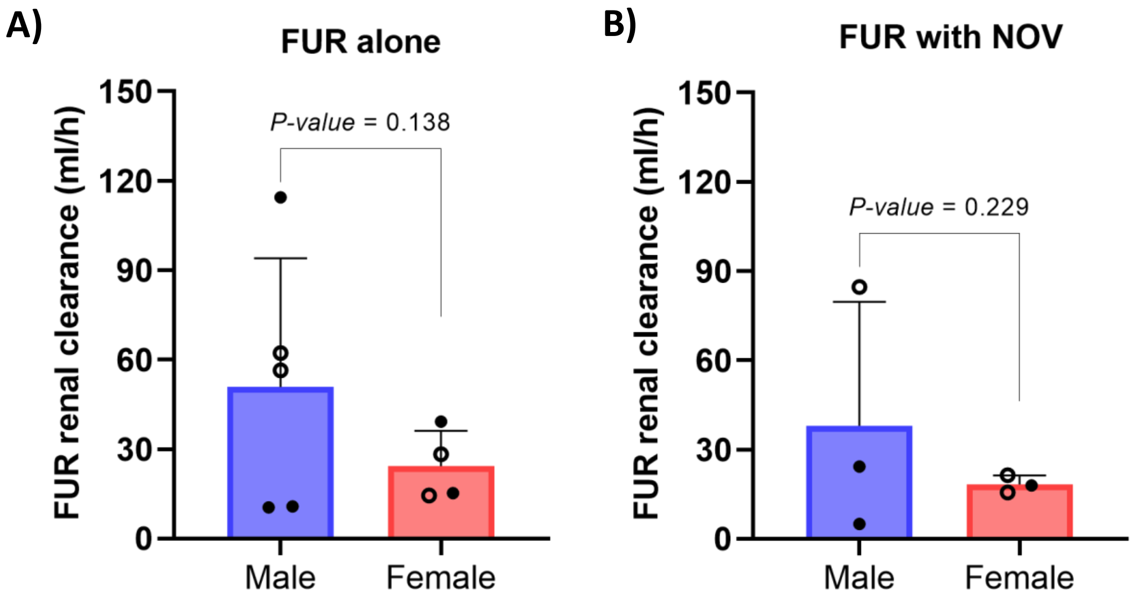

Figure S5. Renal clearance of furosemide (FUR) alone (A), and with novobiocin (NOV) (B), calculated using mid-point approach ($CL_R = \frac{Ae_{0-3}/\Delta t}{C_{1.5}}$), where CL_R is renal clearance; Ae_{0-3} is the cumulative amount of drug excreted unchanged in the urine between 0-3 h; Δt is change in time; and $C_{1.5}$ is FUR blood concentration at the mid-point of the urine collection interval (i.e., 1.5 h). The renal clearance estimation based on the AUC_{0-6} (Figure 4) and mid-point methods showed similar trend on the effect of sex on renal clearance. Because no food-effect was observed, fed (closed circle) and fasted (open circle) data for the male and female rats were included as separate data points. The symbols represent individual data points. Data were compared by unpaired Student's t-test (*P-value* > 0.05 was considered not significant).
